# Health Tract, No. 26

**Published:** 1865

**Authors:** 


### HEALTH TRACT, No. 26.

---

### EATING.

The stomach has two doors, one for the entrance of the food, on the left side, the other, for its exit, after it has been properly prepared for another process. As soon as the food is swallowed, it begins to go round and round the stomach so as to facilitate dissolution; just as the melting of a number of small bits of ice is expedited by being stirred in a glass of water; the food, like the ice, dissolving from without, inwards, until all is a liquid mass.

Eminent physiologists have said, that as this liquid mass passes the door of exit, where there is a little movable muscle, called the Pyloric Valve, (a faithful watchman,) that which is fit for future purposes gives a tap, as it were; the valve flies open, and it makes an honorable exit. Thus it goes on until the stomach is empty, provided no more food has been taken than there was a supply of gastric juice for. If a mouthful too much has been taken, there is no gastric juice to dissolve it; it remains hard and undigested, it is not fit to pass, and the janitor refuses to open the door; and another and another circuit is made, with a steady refusal at each time, until the work is properly done. Boiled rice, roasted apples, cold raw cabbage cut up fine in vinegar, tripe prepared in vinegar, or souse, pass through in about an hour; fried pork, boiled cabbage and the like, are kept dancing around for about five hours and a half.

After, however, there has been a repeated refusal to pass, and it would appear that any longer detention was useless, as in the case of indigestible food, or a dime, or cent, or fruit-stone, the faithful watchman seems to be almost endowed with intelligence as if saying: "Well, old fellow, you never will be of any account; it is not worth while to be troubled with you any longer, pass on, and never show your face again."

When food is thus unnaturally detained in the stomach, it produces wind, eructations, fullness, acidity, or a feeling often described as a "weight," or "load," or "heavy." But nature is never cheated. Her regulations are never infringed with impunity; and although an indigestible article may be allowed to pass out of the stomach, it enters the bowels as an intruder, is an unwelcome stranger, the parts are unused to it, like a crumb of bread which has gone the wrong way by passing into the lungs, and nature sets up a violent coughing to eject the intruder. As to the bowels, another plan is taken, but the object is the same—a speedy riddance. As soon as this unwelcome thing touches the lining of the bowels, nature becomes alarmed, and like as when a bit of sand is in the eye, she throws out water, as if with the intention of washing it out of the body, hence the sudden diarrheas with which two-legged pigs are sometimes surprised. It was a desperate effort of nature to save the body, for if undigested food remains too long, either in the stomach or bowels, fits, convulsions, epilepsies, apoplexies, and death, are a very frequent result. Inference: *Always eat slowly and in moderation of well-divided food.*

